# Fluoxetine and thioridazine inhibit efflux and attenuate crystalline biofilm formation by *Proteus mirabilis*

**DOI:** 10.1038/s41598-017-12445-w

**Published:** 2017-09-22

**Authors:** Jonathan Nzakizwanayo, Paola Scavone, Shirin Jamshidi, Joseph A. Hawthorne, Harriet Pelling, Cinzia Dedi, Jonathan P. Salvage, Charlotte K. Hind, Fergus M. Guppy, Lara M. Barnes, Bhavik A. Patel, Khondaker M. Rahman, Mark J. Sutton, Brian V. Jones

**Affiliations:** 1 School of Pharmacy and Biomolecular Sciences, University of Brighton, Lewes Road, Brighton, BN2 4GJ United Kingdom; 2 Department of Microbiology, Instituto de Investigaciones Biológicas Clemente Estable, Montevideo, CP 11600 Uruguay; 3 Institute of Pharmaceutical Science, King’s College London, 150 Stamford Street, London, SE1 9NH United Kingdom; 4 National Infections Service, Public Health England, Porton Down, Salisbury, SP4 0JG United Kingdom

## Abstract

*Proteus mirabilis* forms extensive crystalline biofilms on indwelling urethral catheters that block urine flow and lead to serious clinical complications. The *Bcr/CflA* efflux system has previously been identified as important for development of *P. mirabilis* crystalline biofilms, highlighting the potential for efflux pump inhibitors (EPIs) to control catheter blockage. Here we evaluate the potential for drugs already used in human medicine (fluoxetine and thioridazine) to act as EPIs in *P. mirabilis*, and control crystalline biofilm formation. Both fluoxetine and thioridazine inhibited efflux in *P. mirabilis*, and molecular modelling predicted both drugs interact strongly with the biofilm-associated *Bcr/CflA* efflux system. Both EPIs were also found to significantly reduce the rate of *P. mirabilis* crystalline biofilm formation on catheters, and increase the time taken for catheters to block. Swimming and swarming motilies in *P. mirabilis* were also significantly reduced by both EPIs. The impact of these drugs on catheter biofilm formation by other uropathogens (*Escherichia coli*, *Pseudomonas aeruginosa*) was also explored, and thioridazine was shown to also inhibit biofilm formation in these species. Therefore, repurposing of existing drugs with EPI activity could be a promising approach to control catheter blockage, or biofilm formation on other medical devices.

## Introduction

Indwelling urethral catheters (IUC) are widely used for long-term bladder management in both community and nursing home settings, and can provide significant improvement to quality of life for many patients. However, the care of individuals undergoing long-term catheterisation is frequently undermined by acquisition of infection (catheter associated urinary tract infection; CAUTI). *Proteus mirabilis* is a particularly problematic and prevalent pathogen in this regard, and this species is amongst the most commonly isolated from CAUTI^1,2^. Colonisation of the catheterised urinary tract by *P. mirabilis* is also associated with the onset of serious clinical complications, stemming from its ability to form unusual crystalline structures on catheter surfaces, which can damage tissues and block urine flow^1–3^.

The encrustation and blockage of catheters by *P. mirabilis* is attributable to its ability to form extensive biofilm communities on catheter surfaces, coupled with the production of a potent urease enzyme that hydrolyses urea and forms ammonia^4–7^. The generation of ammonia *via* ureolysis leads to a rapid rise in urinary pH and in the resulting alkaline conditions, normally soluble components of urine, precipitate and form crystals of ammonium magnesium phosphate (struvite) and calcium phosphate (hydroxyapatite)^2,4,7–10^. These crystals become trapped in the growing biofilm matrix and are incorporated into its structure^2,4^. The action of the *P. mirabilis* urease also generates a highly alkaline microenvironment within biofilms, while the exopolymeric matrix encasing biofilm cells can attract and bind calcium and magnesium ions, which collectively serve to accelerate and stabilise biofilm-associated crystal growth^11,12^. Ultimately these processes result in the extensive mineralisation of biofilms, and the development of crystalline biofilm structures which obstruct urine flow^2–4^.

Because the majority of patients undergoing long-term catheterisation are cared for outside the hospital environment where continual clinical monitoring is not available, catheter blockage is often not noticed until more serious consequential complications arise^3^. A particular hazard of catheter blockage is the accumulation of infected urine in the bladder, which eventually results in reflux to the upper urinary tract and subsequent onset of pyelonephritis, septicaemia, and shock^3,13^. It has been estimated that ~50% of individuals undergoing long-term catheterisation will suffer from catheter blockage at some point during their care, with a considerable proportion experiencing repeated and chronic blockage^3,14^. It is perhaps then unsurprising that blockage is also the cause of numerous emergency hospital referrals, and not only damages the health of patients, but also places significant strain on healthcare resources^3,15^.

Although a range of catheter types have been developed in attempts to combat CAUTI, all remain susceptible to *P. mirabilis* encrustation and are of no value in the long-term setting^3,16,17^. These include catheters with antimicrobial coatings such as nitrofurazone and silver, which have been found to be ineffective in preventing CAUTI even during short-term use^18^. Furthermore, once *P. mirabilis* colonises the catheterised urinary tract it is exceedingly difficult to eradicate, and can persist through numerous catheter changes and antibiotic treatments, resulting in chronic infections and recurrent blockage in some patients^19^. Currently, there are no fully effective methods for control of *P. mirabilis* CAUTI and associated blockage, and further work in this area is urgently needed.

Recently, we demonstrated that efflux systems are important in the formation of *P. mirabili*s crystalline biofilms, and *P. mirabilis* mutants with disruptions in the *Bcr/CflA* efflux system (a member of the Major Facilitator Superfamily of transporters) were found to be significantly attenuated in ability to form crystalline biofilms and block catheters^20^. This raises the potential for efflux pump inhibitors (EPIs) to provide a novel and effective approach to control bacterial biofilm formation in the catheterised urinary tract, and combat catheter blockage by *P. mirabilis*. Moreover, licensed drugs already commonly used in human medicine have been shown to have potential EPI activity in other bacterial species, and also to reduce biofilm formation through this activity^21–25^. Here we tested the selective serotonin re-uptake inhibitor, fluoxetine, and the antipsychotic-drug, thioridazine, for potential EPI activity in *P. mirabilis* and interaction with the biofilm-associated *Bcr/CflA* efflux system. Subsequently, the capacity of these drugs to attenuate crystalline biofilm formation was evaluated using a representative model of the catheterised urinary tract.

## Results

### Fluoxetine and thioridazine inhibit efflux in *Proteus mirabilis*

**Figure 1:**
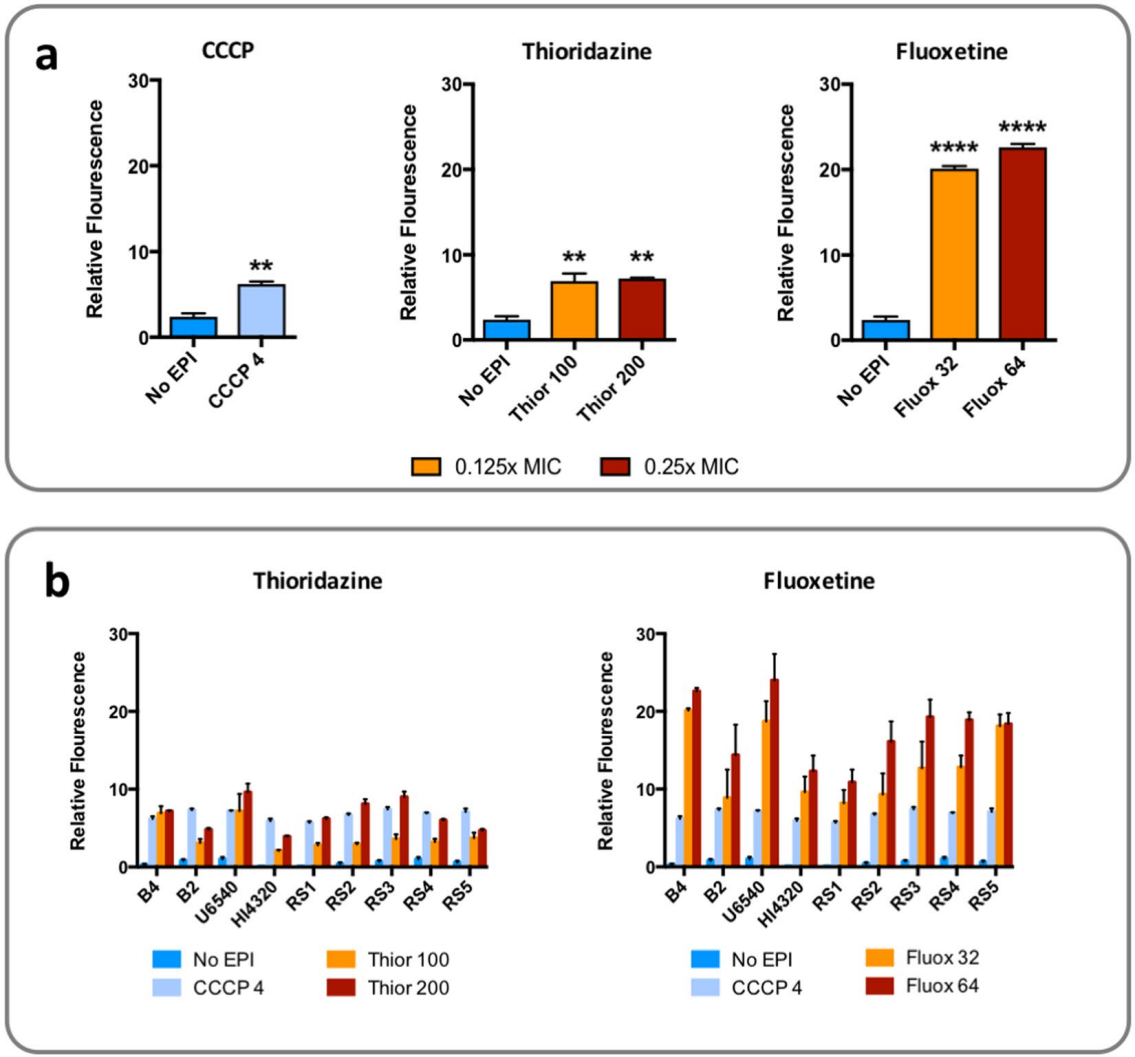
Effect of putative EPIs on accumulation of EtBr in *P. mirabilis*. The EPI activity of thioridazine and fluoxetine was assessed using the EtBr accumulation assay in artificial urine supplemented with 20 mM glucose, against the *P. mirabilis* catheter isolates. Fluorescence intensity relative to cell free controls was measured after 2 h exposure to EPIs at concentrations (µg/mL) reflecting 0.125x and 0.25x the MIC for each drug against *P. mirabilis* strain B4. Readings from treated cells were compared with untreated controls (No EPI but with EthBr substrate), and cells treated with the proton gradient decoupling agent carbonyl cyanide m-chlorophenyl hydrazine (CCCP), as a+ve control for the accumulation assay. (**a**) Results of accumulation assays for treatment with CCCP, thioridazine, or fluoxetine. (**b**) Results of accumulation assays for treatment with CCCP, thioridazine, or fluoxetine against a broader panel of *P. mirabilis* clinical isolates **P < 0.001, ****P < 0.00001 vs untreated controls. All data represent the mean of 3 replicates. Error bars show standard error of the mean.

Fluoxetine and thioridazine were screened for inhibition of efflux in *P. mirabilis* strain B4 using an ethidium bromide (EtBr) accumulation assay. The proton gradient decoupling agent carbonyl cyanide m-chlorophenyl hydrazine (CCCP) was utilised as a general inhibitor of energy-dependent membrane transport, and a positive control for the accumulation assay (Fig. 1). Assessment of minimum inhibitory concentrations (MICs) demonstrated an MIC of 256 µg/mL for fluoxetine, and 800 µg/mL for thioridazine against *P. mirabilis* B4. Efflux assays were subsequently conducted with concentrations reflecting 0.125x and 0.25x the MIC.

At concentrations tested, both drugs exhibited putative efflux inhibition against *P. mirabilis* strain B4, as indicated by increased cellular fluorescence in treated vs. untreated cells in EtBr accumulation assays (Fig. 1a). Fluoxetine generated the greatest increase in cellular fluorescence suggesting a greater level of EtBr efflux inhibition was achieved with this drug compared to thioridazine (Fig. 1a). A dose-dependent effect was observed for fluoxetine, with levels of cellular fluorescence increasing in line with the concentration of the drug tested, but for thioridazine both concentrations tested elicited a comparable increase in cellular fluorescence (Fig. 1a). Broadly similar results were obtained when these drugs were tested against an extended panel of *P. mirabilis* clinical isolates, with fluoxetine consistently producing a greater increase in cell fluorescence compared to thioridazine (Fig. 1b). However, a notable level of strain-strain variability was evident in response to both drugs, and in contrast to strain B4, thioridazine generated much more pronounced dose-dependent changes in cell fluorescence for most other strains tested (Fig. 1b).

### Interaction of thioridazine and fluoxetine with P. mirabilis efflux systems involved in crystalline biofilm formation

**Figure 2:**
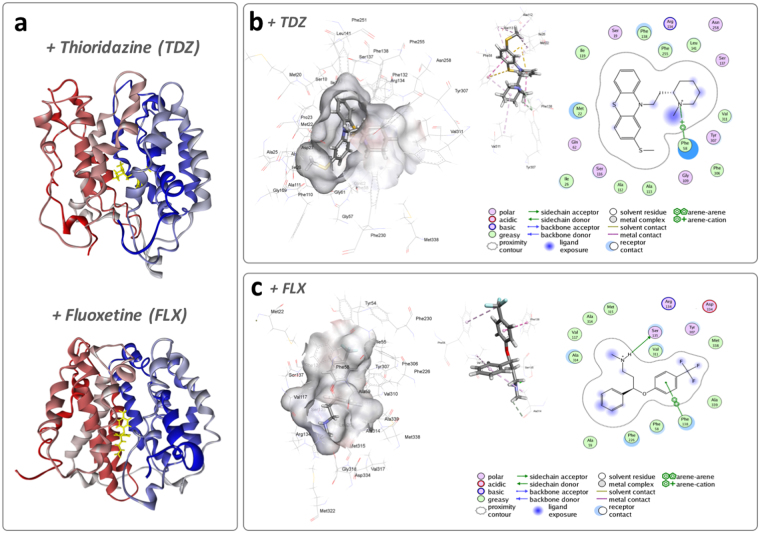
Interaction of fluoxetine and thioridazine with the biofilm-associated *Bcr/CflA* transporter. (**a**) Average 2D and 3D structures of bcr/CflA:thioridazine and bcr/CflA:fluoxetine; Binding sites and key interactions between the *Bcr/CflA* transporter and (**b**) thioridazine; (**c**) fluoxetine.

Using an *in silico* molecular modelling approach, we evaluated the potential for thioridazine and fluoxetine to interact with the *Bcr/ClfA* efflux system, which we have previously shown to be important for *P. mirabilis* crystalline biofilm formation^20^. The blind molecular docking study, in which the ligand could explore the entire structure of the protein to find probable cavities to bind in, showed the ligands bind to the channel region of the transporter (Fig. 2a and Supplementary Figure 4). Interestingly, both ligands occupied binding sites close to each other, suggesting a similar mode of inhibition. GOLD flexible molecular docking showed that the association between the *Bcr/ClfA* protein and fluoxetine or thioridazine are favourable, and that these associations stabilize the complex by about −33.6 and −40 kcal/mol, respectively. Both drugs are predicted to occupy a hydrophobic amino acid rich binding pocket and show strong interaction with Ile26, Phe58, Phe110, Val117, Phe138, Tyr307, Val310, Val311, Val313 and Ala314 (Supplementary Figure 5).

Time dependence of root-mean-square deviations (RMSD) (Å) for the backbone atoms relative to the starting structure during 100 ns MD simulations of both ligand-free and ligand-bound *Bcr/CflA* pump are shown in Supplementary Figure 6. It can be seen from the RMSD curves that the *Bcr/CfIA*-fluoxetine complex showed notably more fluctuations during the first 50 ns of the simulation compared to the *Bcr/CfIA*-thioridazine complex. Both complexes appeared to stabilise and reach a steady state after ~80 ns, indicated by the relatively stable RMSD values from 80 ns to the end of the simulations (Supplementary Figure 6). The average structures of each complex were extracted from the MD trajectories and analyzed to study the molecular level interaction during the simulations. The orientation and the interaction of the compounds with the key residues within the binding site are shown in Fig. 2b,c.

Post MD simulations energy analysis using MM-PBSA/MM-GBSA calculations showed that *Bcr/CflA* -thioridazine, with the energy of binding being −38.6 kcal/mol, formed a more stable and favorable complex compared to the *Bcr/CflA*-fluoxetine, which showed an energy of binding of −26.7 kcal/mol. Comparison between the GB energies, which are calculated in a different way and puts emphasis on the electerostatic components of the energy, still showed a notably better energy of binding for the *Bcr/CflA*-thioridazine complex (−47.74 kcal/mol), compared to the *Bcr/CflA*-fluoxetine complex (−33.95 kcal/mol) (Table 1).

**Table 1:** Average energy contributions to form *Bcr/CflA* complexes fluoxetine or thioridazine.

Interaction	Complex^a^	
	Bcr/CflA-Thioridazine	Bcr/CflA-Fluoxetine
Electrostatic (ΔE_ele_)	−9.08(1.31)	−8.17(1.33)
van der Waals (ΔE_vdw_)	−51.81(1.42)	−36.36(1.99)
Surface energy (ΔE_sur_)	−6.79(0.11)	−5.88(0.10)
Solvation energy (ΔE_sol_)	22.28(2.27)	17.77(1.90)
Polar solvation energy (ΔG_PB_)	−38.60(2.20)	−26.75(2.39)
Electrostatic solvation energy (ΔG_GB_)	−47.74(1.60)	−33.95(2.18)

^a^Interaction energies between complexes of *Bcr/CflA* and either fluoxetine or thioridazine (kcal/mol). Figures in parentheses provide standard error of the mean.

Interaction energies between the ligands and the key residues within the binding site of *Bcr/CflA* obtained from the average structures during the molecular dynamics simulations are listed in the Table 2. The analysis showed that fluoxetine showed strong interactions with Gly57, Met338, Val310, and Val311, while thioridazine showed very strong interactions with Met22, Phe58, Phe138 and Ser116. These amino acid residues mainly show hydrogen bond, Pi-cation, and electrostatic interactions that help to stabilize the complex. The molecular simulation data suggests that these strong interactions can potentially lead to the inhibition of the *Bcr/CflA* pump.

**Table 2:** The interaction energies between fluoxetine or thioridazine and important key residues of ligand binding site in average structures *Bcr/CflA*-ligand complexes.

Complex^a^	Residue^b^	Interaction energy (kJ/mol)^c^
Bcr/CflA-Thioridazine	**Ala 113**	−10.64
	**Arg 134**	−14.10
	Gln 62	−10.23
	Met 22	−24.36
	Phe 58	−46.44
	Phe 138	−22.06
	Phe 255	−6.97
	Phe 306	−6.36
	Pro 23	−4.33
	Ser 19	−9.12
	**Ser 116**	−13.40
	**Val 311**	−7.18
Bcr/CflA-Fluoxetine	**Ala 113**	−10.61
	Ala 114	−10.68
	**Arg 134**	−10.10
	Gly 57	−13.58
	Ile 55	−11.63
	Met 338	−15.03
	Phe 56	−10.79
	**Ser 116**	−7.02
	Tyr 54	−8.91
	Tyr 307	−7.62
	Tyr 335	−9.50
	Val 310	−16.84
	**Val 311**	−22.41

^a^Average structures of complexes of *Bcr/CflA* and either thioridazine or fluoxetine were extracted from 100 ns molecular dynamics trajectories. ^b^Residues derived from average structures. Residues in bold denote those identified in both thioridazine and fluoxetine models. ^c^Interaction energies between residues in average structures of *Bcr/CflA*-ligand complexes.

### Effect of EPI treatment on *P. mirabilis* crystalline biofilm formation, pH elevation, and survival in bladder models

**Figure 3:**
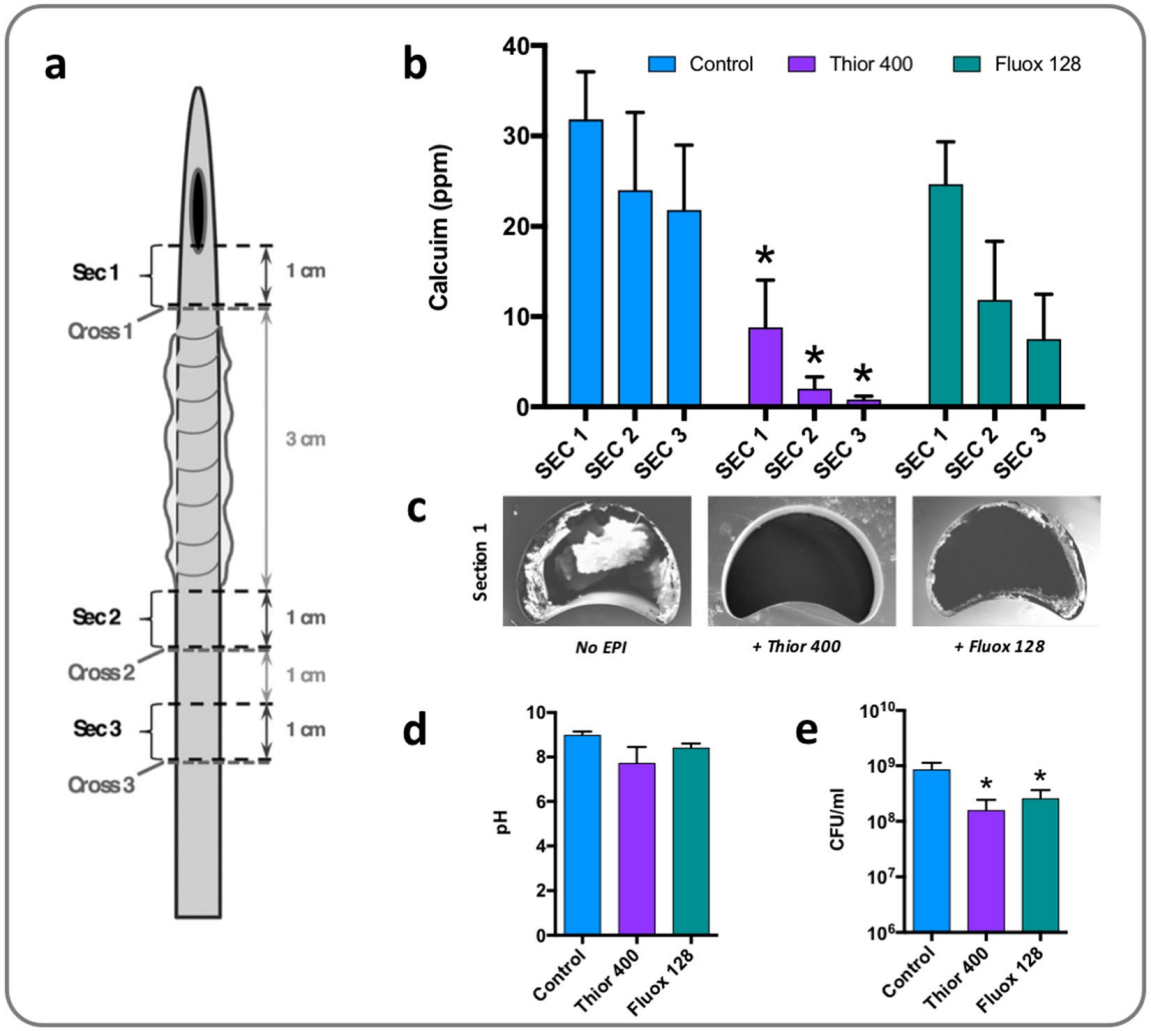
Effect of EPI administration on *P. mirabilis* crystalline biofilm formation. To investigate the impact of EPI treatment on crystalline biofilm formation, timed bladder model experiments were conducted and levels of encrustation quantified and visualised at the end of experiments. Models were supplied with standardised artificial urine with initial pH of 6.1, and containing EPIs at 0.5x MIC of each drug tested (thioridazine 400 µg/mL, or fluoxetine 128 µg/mL). Models were inoculated with 10^9^ CFU/mL of *P. mirabilis* strain B4 (simulating established infection), and viable planktonic cells and pH measured in residual bladder after 10 h when models were deactivated. Catheters were removed and sectioned for analysis at the 10 h time point. (**a**) Schematic showing catheter sections analysed for levels of encrustation. (**b**) Quantification of calcium on catheter sections by flame photometry as a measure of encrustation. (**c**) Scanning Electron Microscopy of cross sections adjacent to catheter section 1 showing levels of crystalline biofilm formed in control and treated catheters. (**d**) pH of residual bladder urine after 10 h. (**e**) Number of viable cells present in residual urine after 10 h. Data represent a minimum of three replicate experiments. Error bars show standard error of the mean. *P < 0.05 vs control. The diagram of catheter section used in part a of this Figure is taken from Holling *et al*. 2014 *Infect Immun* 80: 1616–1626^20^.

To determine if exposure to thioridazine or fluoxetine could significantly reduce *P. mirabili*s crystalline biofilm formation, and identify any effects on elevation of urinary pH or bacterial viability in the model system, these drugs were tested using *in vitro* infection models of the catheterised urinary tract (Supplementary Figure 1; Stickler *et al*.^26^). Models simulating an established high-level infection (10^9^ CFU/mL) were run for a set time (10 h) and levels of crystalline biofilm formation in treated models compared with untreated controls (Fig. 3). A significant reduction in encrustation of catheters was evident in models treated with thioridazine (Fig. 3b). In contrast, no statistically significant reduction in encrustation was observed for catheters taken from fluoxetine-treated models over this length of time, although a general reduction was noted on distal sections compared to untreated controls (Fig. 3b). Direct visualisation of crystalline biofilm formation by low-vacuum SEM of catheter cross sections were in line with the results of calcium quantification, and also demonstrated reduced levels of crystalline deposits for EPI treated catheters, with a marked absence of encrustation on thioridazine treated catheters (Fig. 3c). The elevation of urinary pH was found to be unaffected by EPI treatment in these timed model experiments (Fig. 3d), but numbers of viable *P. mirabilis* cells in residual bladder urine showed a significant reduction in models treated with both drugs (Fig. 3e).

### Impact of EPI treatment on catheter blockage

To determine if effects on crystalline biofilm formation by fluoxetine or thioridazine translated to a significant extension of catheter life span and a delay in catheter blockage, we measured the time taken for catheters to become blocked in *in vitro* models treated with these drugs, compared to untreated controls. Models were used to simulate scenarios of both established high-level infection (10^9^ CFU/mL) or early colonisation of the catheterised urinary tract (10^3^ CFU/mL) (Nzakizwanayo *et al*.^27^), and doubling concentrations of drugs reflecting 0.125x, 0.25x and 0.5x the *P. mirabilis* B4 MIC were evaluated.

**Figure 4:**
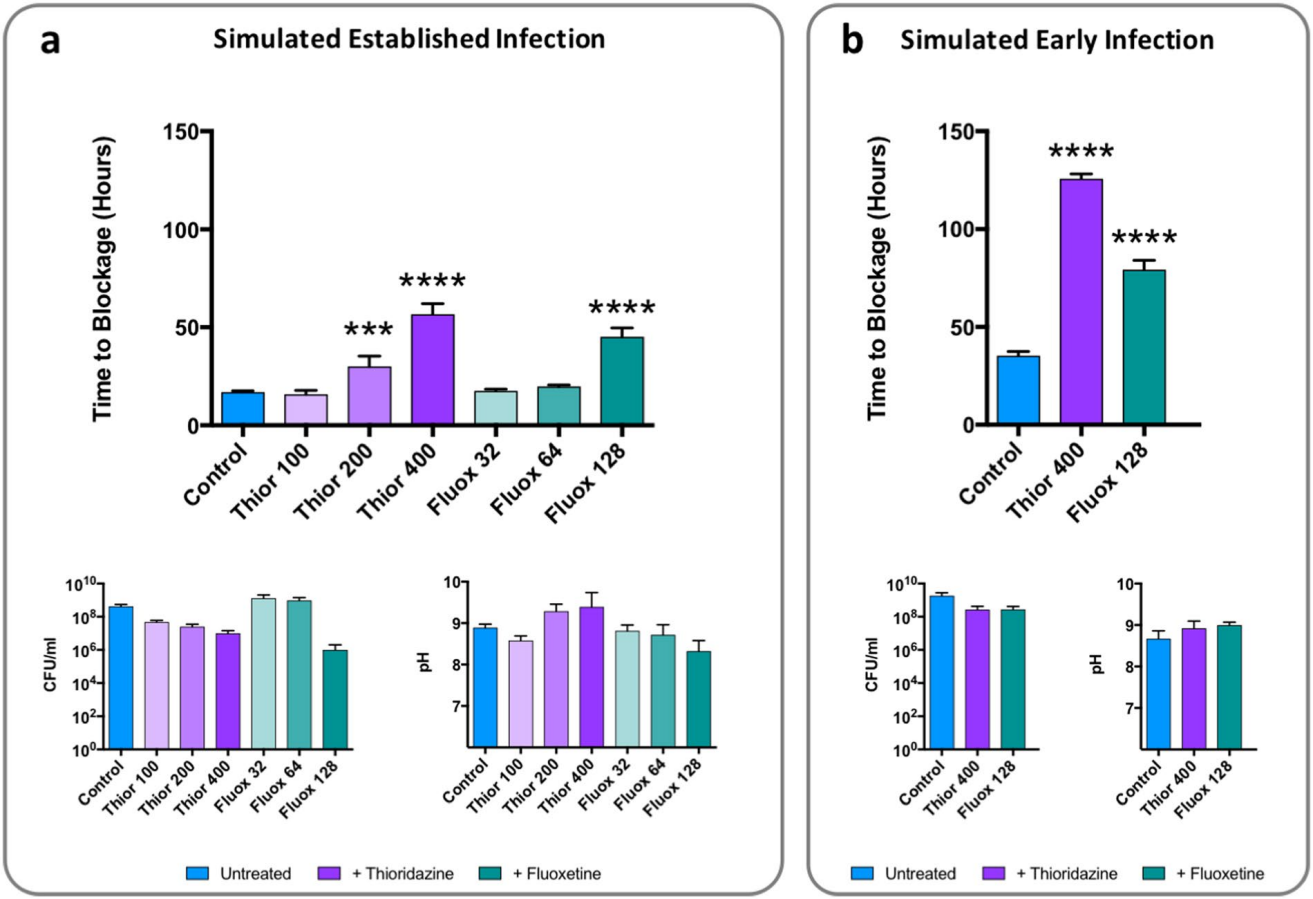
Effect of EPI administration on ability of *P. mirabilis* to block indwelling urethral catheters. Models were supplied with standardised artificial urine with initial pH of 6.1, and EPIs thioridazine and fluoxetine were supplied in artificial urine media throughout experiment. Models simulating both established infection (inoculated with 10^9^ CFU/mL of *P. mirabilis* strain B4), or early stages of infection (inoculated with 10^3^ CFU/mL of *P. mirabilis* strain B4) were run. Upon blockage viable planktonic cells in residual urine in “bladders” were enumerated and pH measured. (**a**) Impact of EPI treatment on time taken for catheters to block in models simulating established infection. Models were treated with concentrations reflecting 0.125x, 0.25x, or 0.5x the MIC of each drug (100, 200, 400 µg/mL thioridazine; 32, 64, 128 µg/mL fluoxetine). (**b**) Impact of EPI treatment on time taken for catheters to block in models simulating early stages of infection. Models were treated with concentrations reflecting 0.5x MIC values for each drug (400 µg/mL thioridazine, 128 µg/mL fluoxetine). Data represent a minimum of three replicate experiments. Error bars show standard error of the mean. ***P < 0.0001 vs control; ****P < 0.00001 vs control.

A significant dose-dependent increase in the time taken for catheters to block was observed in thioridazine treated models of established infection (Fig. 4a). Fluoxetine was also observed to generate a statistically significant increase in time to blockage, but this was less pronounced than that observed for thioridazine, and was only evident at the highest concentration tested (0.5x MIC, Fig. 4a). Congruent with these results, treatment of models simulating early low-level colonisation of the catheterised urinary tract with the highest doses of thioridazine or fluoxetine (0.5x bacterial MIC), produced a commensurately greater increase in time to blockage (Fig. 4b). No significant effects of EPI treatment were observed on elevation of urinary pH, or levels of viable cells in residual bladder model urine in either established or early stage models at time of blockage, but a trend towards reduced viable cell counts at time of blockage with increasing drug concentrations was evident in models of established infection treated with thioridazine (Fig. 4a).

### Impact of EPI treatment on swimming and swarming motility

**Figure 5:**
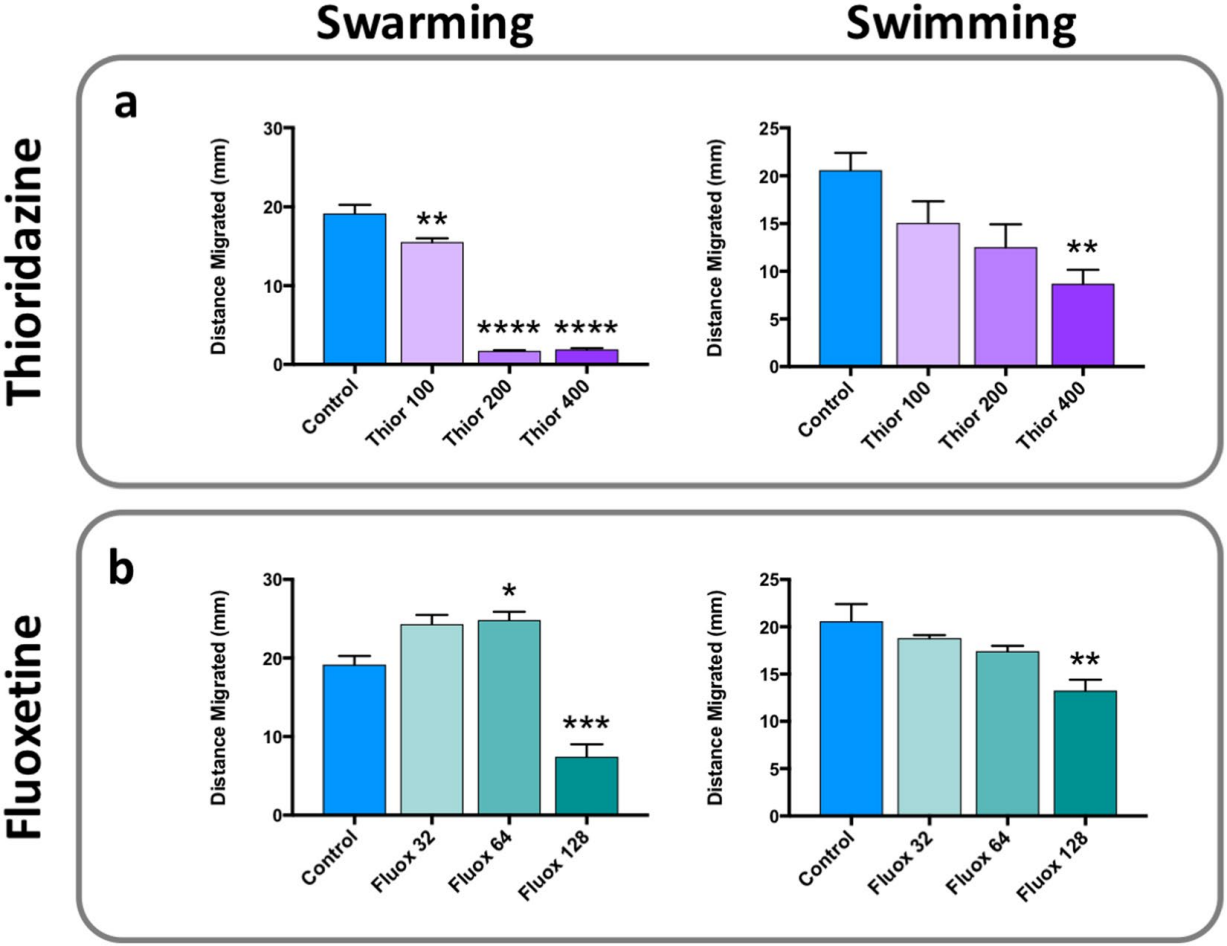
Effect of EPI treatment on motility. The impact of EPI treatment on swimming and swarming motility in strain B4 was evaluated by supplementation of LB agar (1.5% for swarming and 0.15% for swimming) with either (**a**) thioridazine (100, 200 or 400 µg/mL) or (**b**) fluoxetine (32, 64 or 128 µg/mL). Significant differences in motility upon EPI exposure are denoted by changes in distances migrated (mm) compared to untreated controls. Data represent the mean of at least 3 replicates, and error bars show standard error of the mean. *P < 0.05 vs untreated control; **P < 0.001 vs untreated control; ***P < 0.0001 vs untreated control; ****P < 0.00001 vs untreated control.

Because our previous biofilm formation deficient mutant with disruption in the *Bcr/CflA* efflux system also displayed considerable reduction in motility, we also assessed the impact of thioridazine and fluoxetine treatment on swimming and swarming in *P. mirabilis*. Thioridazine exhibited the greatest impact on both motilities, with concentrations at 0.25x and 0.5x MIC (200 and 400 µg/mL respectively), almost completely abolishing swarming whilst swimming motility was reduced in a dose-dependent manner (Fig. 5a). In contrast, fluoxetine had less effect on both swimming and swarming at the concentrations tested. A dose-dependent decrease in swimming motility was evident with increasing concentrations of fluoxetine, but while a significant reduction in swarming was noted at 0.5x MIC (128 µg/mL), lower concentrations appeared to increase swarming motility (Fig. 5a).

### Impact of thiroidazine and fluoxetine treatment on catheter biofilm formation by other uropathogens

**Figure 6:**
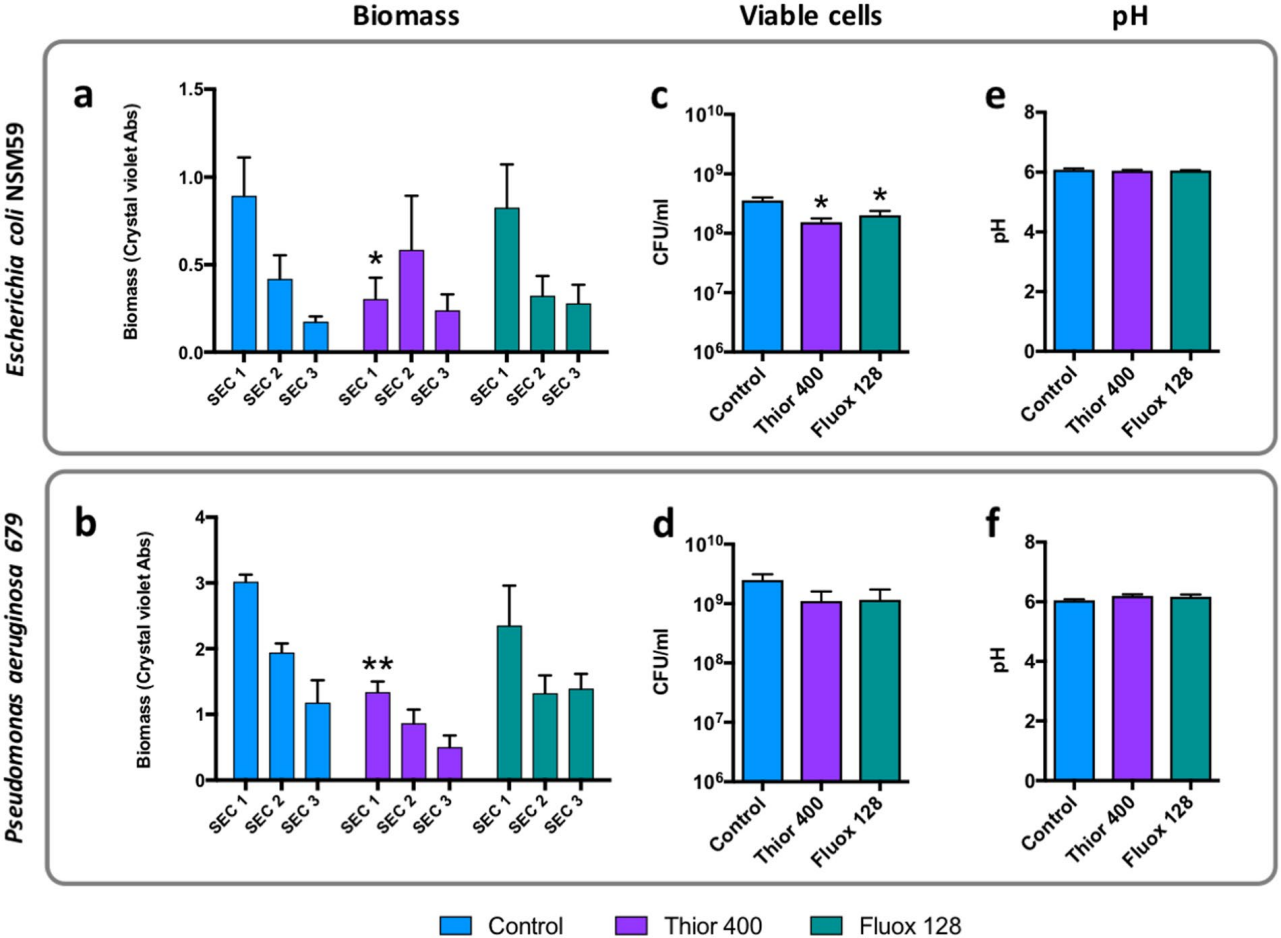
Effect of EPI administration on catheter biofilm formation by other uropathogens. To investigate the impact of EPI treatment on biofilm formation *by Escherichia coli* and *Pseudomonas aeruginosa*, timed bladder model experiments were conducted and levels of biofilm formation quantified at the end of experiments. Models were supplied with standardised artificial urine with initial pH of 6.1, and containing EPIs at 0.5x MIC of each drug tested (thioridazine 400 µg/mL, or fluoxetine 128 µg/mL). Models were inoculated with 10^9^ CFU/mL of *E. coli* or *P. aeruginosa* (simulating established infection), and viable planktonic cells and pH measured in residual bladder after 10 h when models were deactivated. Catheters were removed and sectioned for analysis at the 10 h time point as shown in Fig. 6. (**a**,**b**) Quantification of biomass on catheter sections using modified crystal violet biofilm quantification assay. (**c**,**d**) Number of viable cells present in residual urine after 10 h. (**e**,**d**) pH of residual bladder urine after 10 h. Data represent a minimum of three replicate experiments. Error bars show standard error of the mean. *P < 0.05, ** < 0.01 vs control.

Because biofilm formation is also a key feature of infections caused by other urinary tract pathogens relevant to CAUTI, we also evaluated the potential for thioridazine or fluoxetine to control catheter biofilm formation in two other common uropathogens, *Escherichia coli* and *Pseudomonas aeruginosa*, when deployed at concentrations inhibiting *P. mirabilis* efflux and crystalline biofilm formation. Because these species do not encrust or block catheters, the impact of EPI treatment was evaluated in timed bladder model experiments (simulating established infection), and levels of biofilm formation assed by a modified crystal violet assay (Fig. 6). In both species thioridazine treatment was able to produce a significant reduction in catheter biofilm on sections of catheter proximal to the eye-hole, where the greatest levels of biofilm formation typically occur (Fig. 6a,b). In contrast, no significant reduction in biofilm formation was elicited by fluoxetine treatment (Fig. 6a,b). Both thioridazine and fluoxetine produced a small but statistically significant reduction in numbers of viable *E. coli* cells in bladder models at concentrations tested, but no similar significant effects were observed for *P. aeruginosa* (Fig. 6c,d). No EPI tested had any influence on pH of artificial urine in these experiments (Fig. 6e,f).

## Discussion

We have previously linked efflux activity to crystalline biofilm formation and motility in *P. mirabilis*, and demonstrated the importance of the *Bcr/CflA* MFS transporter to catheter blockage by this organism^20^. Results obtained in this study now provide further evidence that efflux systems are important to the formation of crystalline biofilms by *P. mirabilis*, and confirm that suppression of efflux using chemical inhibitors is a potentially viable approach to control biofilm formation in the catheterised urinary tract. The molecular docking study, followed by molecular dynamic simulations, also suggests that both drugs evaluated here (fluoxetine and thioridazine) bind favorably within the channel region of the biofilm-associated *Bcr/CflA* transporter, and form stable complexes. These interactions are predicted to be strong enough to inhibit the functional mechanism of the transporter, and when considered in the context of our previous studies of biofilm formation in *Bcr/CflA* transposon mutants^20^, are in keeping with the reduction in crystalline biofilm formation observed in bladder models treated with fluoxetine or thioridazine.

The overall findings of this study are also congruent with a growing body of evidence supporting a role for efflux systems in numerous aspects of bacterial virulence, including in pathogens relevant to CAUTI such as *P. aeruginosa*, *E. coli, Staphylococcus aureus*, and *Klebsiella pneumoniae*. These include aspects of survival and fitness within the host, ability to initiate infection, survival and replication within host cells, roles related to the expression of virulence attributes, and also biofilm formation^20,21,23,24,28–36^. Perhaps of most relevance to this study are investigations of biofilm formation in *E. coli* and *P. aeruginosa*, which have identified genes encoding efflux systems to be up-regulated during biofilm formation (compared to planktonic cells), among the most highly expressed genes in mature biofilms, and have linked the reduced antibiotic susceptibility of biofilms to over expression of efflux in these communities^23,28,37–39^. An EPI-mediated reduction in biofilm formation, including through the use of thioridazine, has also previously been reported in *E. coli* ^23^. Collectively these previous studies fit well with the mitigation of biofilm formation we also observed in *E. coli* and *P. aeruginosa* with thioridazine treatment.

Recent investigations have also pointed to a range of potential mechanisms through which inhibition of efflux may impact on biofilm formation. For example, signalling molecules which mediate cell-cell communication, and regulate population density dependent processes are substrates for some efflux systems^33,40,41^. This opens the possibility for efflux inhibitors to interfere with quorum-sensing and in turn disrupt processes orchestrated by this inter-cellular communication system, including biofilm formation. In addition, studies of *Pseudomonas syringae* demonstrate how the accumulation of normally extruded substrates within the cell, resulting from loss of a particular efflux pump, can alter expression of unexpected gene sets leading to changes in disparate phenotypes^42^. Collectively these observations afford the potential for efflux pumps to contribute to regulation of a wide range of traits relevant to virulence and biofilm formation, and indicate that these transporters may perform regulatory functions beyond their basic accepted role in cellular detoxification.

Alternatively, it has been proposed that some efflux pumps act as a waste management system during maturation of the biofilm^23^, allowing the densely packed population of biofilm-associated cells to deal with the inevitable build-up of toxic metabolic end products as the biofilm expands. Our previous data from a *P. mirabilis Bcr/ClfA* MFS transporter mutant are well aligned with this theory, and disruption of the *Bcr/CflA* system led to defects and reductions in later stages of biofilm development and expansion, but did not appear to impede early events in this process^20^. While the present study did not specifically examine the effects of thioridazine or fluoxetine on distinct stages of biofilm formation, data from timed 10 h bladder model experiments are compatible with potential effects on biofilm maturation and expansion, and EPI treatments generated similar results to those obtained from the defined *Bcr/CflA* mutant in analogous experiments^20^. Nevertheless, further investigation will be required to understand how efflux inhibition leads to reduced crystalline biofilm formation in *P. mirabilis*, and currently available data do not presently rule out any of the mechanisms described above.

Swarming is a complex multicellular behaviour in *P. mirabilis*, sharing many characteristics of biofilm formation, and also likely to be regulated by quorum sensing^43^. Given the almost complete abolition of swarming observed in our previous *Bcr/CflA* efflux mutant^20^, the strong effects of thioridazine and fluoxetine on swarming motility are in keeping with the inhibition of efflux by these drugs, and their predicted interaction with the *Bcr/CflA* system. The effects of thioridazine and fluoxetine on swimming motility are also congruent with the considerable reductions in swimming ability observed in our *P. mirabilis bcr/ClfA* mutant^20^. Moreover, reductions in both swimming and swarming have been noted in other species when efflux systems are disrupted, and linked to aberrations in flagella biosynthesis, and reductions in expression of genes related to motility^30,34,42^. Although the impact of thioridazine or fluoxetine treatment on flagella production in *P. mirabilis* is yet to be determined, this is in keeping with reductions in both swimming and swarming motilities when efflux systems are inhibited, and it is a plausible hypothesis that these drugs may influence flagellar production in *P. mirabilis* as well.

In the context of catheter blockage, both swimming and swarming motilities have been linked with biofilm formation and virulence in several uropathogens^44,45^, and are proposed to contribute to *P. mirabilis* crystalline biofilm formation and CAUTI. However, no concrete role for either motility has yet been established in *P. mirabilis* crystalline biofilm formation specifically, and mutants with defects in motility did not exhibit reduced biofilm formation in bladder model experiments^5^. While this does not fully preclude the potential for reductions in swimming or swarming to play a part in the attenuation of blockage generated by thioridazine and fluoxetine, it seems unlikely that this is a major factor in the effect of these drugs on crystalline biofilm formation. Nevertheless, motility and particularly swarming is considered to be important in the initial colonisation of the catheterised urinary tract^5,43^, and interventions that can suppress motility in *P. mirabilis* may have applications in preventing colonisation of the catheterised urinary tract.

In contrast to swimming and swarming, the ability to grow and persist under the conditions encountered in the catheterised urinary tract, are undoubtedly critical to the pathogenesis of *P. mirabilis* CAUTI, and the formation of crystalline biofilms. Although thioridazine and fluoxetine treatment showed no statistically significant impact on the numbers of viable cells in bladder models at the end of time-to-blockage experiments, a trend towards reduction in viable cell counts was observed as concentrations of thioridazine increased. Moreover, in timed models where responses to treatment were evaluated after 10 h, statistically significant reductions in viable cells were evident in both fluoxetine and thioridazine treated models.

The reduced cell numbers observed at earlier stages of colonisation or infection in bladder models suggest thioridazine and fluoxetine treatment may also delay blockage by impairing *P. mirabilis* growth in the bladder model system. Although this would seem to conflict with use of these drugs at concentrations well below the established MICs (0.5x and below), these MIC values were defined in standard LB broth cultures, and not the specific conditions *P. mirabilis* must cope with in bladder models. Furthermore, only relatively small impacts on growth in bladder models were observed at the concentrations of thioridazine and fluoxetine used in these experiments, which is compatible with use at sub-MIC concentrations.

Overall the inhibition of efflux by thioridazine and fluoxetine appears to perturb a range of traits and processes relevant to *P. mirabilis* CAUTI and catheter blockage, and has comparable effects to the loss of the *Bcr/CflA* transporter on motility and ability to encrust urethral catheters. However, it should be noted that although our molecular modelling predicts these drugs interact strongly with, and can potentially inhibit, the biofilm-associated *Bcr/CflA* transporter, and the effect of fluoxetine and thioridazine treatment generate phenotypes comparable to disruption of the Bcr/CflA gene, the effects of chemical EPIs are not certain to be restricted to a single specific pump, and there is much potential for the results obtained in this study to originate from a broader, more generalised interference with the *P. mirabilis* “effluxome”. In addition, the possibility that these drugs may also have effects on *P. mirabilis* unrelated to efflux inhibition cannot yet be fully excluded. Nonetheless, these data provide further support for efflux systems as a viable target for control of bacterial biofilm formation, and the *Bcr/CflA* MFS transporter in *P. mirabilis* in particular.

Further research will not only need to provide greater understanding of the specific transporters and their substrates affected by thioridazine and fluoxetine, but also how such EPIs may best be utilised to develop strategies for control of CAUTI, or wider bacterial biofilm formation. The use of these drugs according to standard current clinical practice and prescribing approaches is unlikely to be feasible or useful for the control of CAUTI (for reasons relating to safety, efficacy, and pharmacokinetics), and the concentrations utilized in these laboratory studies are much higher than those normally achieved in body fluids such as urine in practice^46–49^. Nevertheless, there is considerable scope to explore localised delivery in the catheterized urinary tract, the use of combinations of EPIs, or the synergistic use of EPIs with antibiotics, which may yield strategies to deploy these drugs for CAUTI control. Moreover, regardless of the potential to utilize these drugs directly, they can nevertheless serve as valuable lead compounds to stimulate the development of new classes of anti-biofilm agents with novel modes of action.

## Materials and Methods

### Bacterial strains, media, and routine culture

#### General culture and media

Clinical isolates of *Proteus mirabilis*, *Escherichia coli*, and *Pseudomonas aeruginosa* from urinary tract infections used in this study were obtained from the Royal Sussex County Hospital, Bristol Southmeads Hospital, and the *P. aeruginosa* isolate from a collection described by De Soyza *et al*.^50^. All chemicals, reagents, and growth media were obtained from Fisher Scientific (United Kingdom), Sigma (United Kingdom), Oxoid (United Kingdom), or Tocris Bioscience (United Kingdom), unless otherwise stated. Bacteria were routinely cultured in Luria-Bertani (LB) medium (5 g/L yeast extract, 10 g/L tryptone, 10 g/L sodium chloride) at 37 °C with shaking or on LB solidified by the addition of 15 g/L technical agar. For the isolation of single colonies of *P. mirabilis* and the suppression of swarming motility, strains were grown on LB agar without salt and 20 g/L technical agar.

#### Artificial urine

The artificial urine (AU) medium previously described by Stickler *et al*.^26^, was initially prepared as a 5x concentrated stock solution containing sodium disulfate (11.5 g/L), magnesium chloride (hexahydrate) (3.25 g/L), sodium chloride (23 g/L), trisodium citrate (3.25 g/L), sodium oxalate (0.1 g/L), potassium dihydrogen orthophosphate (14 g/L), potassium chloride (8 g/L), ammonium chloride (5 g/L), calcium chloride dihydrate (3.25 g/L), urea (125 g/L), gelatin (25 g/L), and tryptone soya broth (5 g/L). Stock solutions of urea and calcium chloride dihydrate were sterilized separately using a Nalgene Vacuum Filter System (0.45 μm nitrocelullose membrane; Sartorius, United Kingdom), while other components were sterilized by autoclaving. For use in bladder models, all components were combined and diluted to 1x strength using sterile deionized water, with the final pH adjusted to 6.1.

### Minimum inhibitory concentrations (MICs) of thioridazine and fluoxetine

The minimum concentrations of fluoxetine and thioridazine capable of inhibiting *P. mirabilis* growth were determined using a broth micro-dilution method. Wells containing LB broth with doubling dilutions of each drug were inoculated with 1 × 10^5^ CFU/mL log phase *P. mirabilis* cells, and incubated statically for 20 h at 37 °C. Growth was determined spectrophotometrically measuring the optical density at 600 nm (OD_600_), and the MIC was defined as the lowest concentration of compound that inhibited measurable growth.

### Efflux inhibition assays

Assays for inhibition of efflux were modified from those originally described by Coldham *et al*.^51^. Overnight LB broth cultures were re-suspended in fresh broth at 1:100 dilution, and grown for a further 1.5 h with shaking. The cells were harvested by centrifugation, re-suspended in AU medium and adjusted to an OD_600_ of 0.6. The adjusted cell suspension was dispensed into a 96-well plate (50 µL/well) containing 50 µL AU supplemented with glucose (40 mM), Ethidium Bromide (EtBr; 10 µg/mL) as an efflux substrate, and sub-MIC levels of thioridazine or fluoxetine. Controls included background control with cell-free AU but with all other supplements and EPIs; cell suspensions with EtBr substrate but no EPI; and cell suspensions with EPIs but no EtBr substrate. Plates were sealed with breathable membranes to prevent evaporation and incubated at 37 °C with gentle rocking for 2 h. Controls to measure the highest accumulation of EtBr in each strain were also set up and consisted of 200 μL of each strain heated to 65 °C for 5 min in 1.5 mL centrifuge tubes to lyse the cells. Following incubation, fluorescence was measured at 540 nm excitation and 600 nm emission and relative accumulation of EtBr (indicative of efflux inhibition) was calculated as: Fluorescence in EPI treated cells - fluorescence in background controls.

### *In vitro* models of the catheterized urinary tract

Bladder models were conducted as originally described by Stickler *et al*.^26^, with minor modifications, and are illustrated in Figure S1. Models consist of a double-walled glass chamber (the bladder) maintained at 37 °C by a water jacket supplied from a circulating water bath. Size 14 French all-silicone Foley catheters (Bard, United Kingdom) were inserted into the bladder and retention balloons inflated with 10 mL sterile water. The catheter was attached to a drainage bag to form a sterile, closed drainage system. AU medium supplemented with sub-MIC levels of EPIs was supplied to the bladder at a constant flow rate of 0.75 mL/min. Bladder models were inoculated with 10 mL of a bacterial culture containing ∼10^9^ CFU/mL and bacterial cells were allowed to establish within the model for 1 h before flow was activated. The number of viable cells in the bladder residual urine and the pH of the medium were measured at the start and end of experiments.

### Molecular modelling methods

#### Homology modelling

The structure of the *Bcr/CflA* efflux pump protein was generated by homology modelling using the I-TASSER webserver. The FASTA uniport code B4ET96 of the amino acid sequence was applied, and the model with the highest C-score and TM-score was selected for the modelling study. The C-score and TM score for the generated model were 1.58 and 0.94, respectively. After homology modelling, the PDB structure was amended in the missing parts by Accelrys discovery studio 4.5 software (Figure S2). Phyre2 (Protein Homology/analogY Recognition Engine V 2.0) was applied to provide a residue-wise profile, to assess the accuracy of the homology model (Figure S3).

#### Molecular docking

Molecular docking protocols are methods which predict the preferred orientation of a bound ligand to a target that forms a stable complex^52,53^. Knowledge of the preferred orientation may in turn be used to predict the strength of association or binding affinity between two molecules using, for example, scoring functions. AutoDock smina^54^, which uses the AutoDock Vina scoring function by default, was used for the blind molecular docking of the ligands thioridazine and fluoxetine to the *Bcr/CflA*, for finding the best binding site by exploring all probable binding cavities of the proteins. SIMNA was performed with default settings, which samples nine ligand conformations using the Vina docking routine of stochastic sampling. Then GOLD molecular docking^55,56^ was applied for the docking of thioridazine and fluoxetine to the SIMNA-located best binding site of the transporter, for performing flexible molecular docking. Based on the fitness function scores and ligand binding positions, the best-docked poses for the ligands were selected. The best docked pose has the highest fitness function score and the most negative energy.

The GOLD molecular docking procedure was performed by applying the GOLD protocol^56^ in the Accelrys Discovery Studio software. The Genetic Algorithm (GA) was used in GOLD ligand docking software to examine thoroughly the ligand conformational flexibility, along with the partial flexibility of the protein^57^. The maximum number of runs was set to 20 for the ligand, and the default parameters selected were 100 population size, 5 for the number of islands, 100,000 number of operations and 2 for the niche size. Default cut-off values of 2.5 Å (dH-X) for hydrogen bonds and 4.0 Å for the van-der-Waals distance were applied. When the top solutions attained the RMSD values within 1.5 Å, the GA docking was terminated.

#### Molecular dynamics (MD) simulations

After the molecular docking, 100-ns independent molecular dynamics simulations were performed for the complexes and the ligand-free protein, which were followed by MM-PBSA/MM-GBSA calculations. All the MD simulations were carried out using the AMBER 12.0 package. Each system was solvated by using an octahedral box of TIP3P water molecules. Periodic boundary conditions and the particle-mesh Ewald (PME) method were employed in the simulations^58^. The Particle Mesh Ewald (PME) method enabled us to calculate the ‘infinite’ electrostatics without truncating the parameters. During each simulation, all bonds in which the hydrogen atom was present were considered fixed, and all other bonds were constrained to their equilibrium values by applying the SHAKE algorithm^59^. The force field parameters for the ligand were generated by using the ANTECHAMBER module of the AMBER program. A cut-off radius of non-covalent interactions was set to 12 Å for the proteins and complexes. Each minimization and equilibration phase was performed in two stages. In the first stage, ions and all water molecules were minimized for 500 cycles of steepest descent followed by 500 cycles of conjugate gradient minimization. Afterwards, the whole system was minimized for a total of 2500 cycles without restraint, wherein 1000 cycles of steepest descent were followed by 1500 cycles of conjugate gradient minimization. In the second stage, the systems were equilibrated for 500 ps while the temperature was raised from 0 to 300 K, and then equilibration was performed without a restraint for 100 ps while the temperature was kept at 300 K. The sampling of reasonable configurations was conducted by running a 100 ns simulation with a 2 fs time step at 300 K and 1 atm pressure. A constant temperature was maintained by applying the Langevin algorithm, while the pressure was controlled by the isotropic position scaling protocol used in AMBER^60^. The time dependence of root-mean-square deviations (RMSD) (Å) for the backbone atoms relative to the starting structure during 100 ns MD simulations of both ligand-free and ligand-bound *Bcr/CflA* were calculated.

### Swimming and swarming assays

Swarming motility was characterized on plates containing LB agar supplemented with sub-MIC levels of EPIs. A 10 μL drop of an overnight culture was inoculated onto the centre of each plate. The drops were allowed to soak into the agar at room temperature, and then the plates were incubated for 8 h at 37 °C. To assess swimming motility, 2 μL of an overnight culture was stabbed into the centre of soft LB agar motility plates (LB medium solidified with 0.15% agar), and incubated for 6 h at 37 °C. The distances that the bacteria migrated on both types of agar, from the point of inoculation, were measured in mm.

### Quantification of crystalline biofilm formation on catheter sections

The amount of calcium present on catheter sections removed from timed bladder model experiments (10 h) was quantified by flame photometry, as described previously (Holling *et al*.^20^). 1 cm sections of catheter were submerged in 2 mL of a solution of ammonium oxalate and oxalic acid (95% and 5% [vol/vol], respectively, from 0.1 M stock solutions). Submerged sections were subjected to vigorous mixing for 3 min, before incubation at room temperature for 30 min. Catheter sections were then removed, and the remaining mixture centrifuged (3,000 × *g* for 10 min). The resulting supernatants were discarded, and pellets resuspended in 5 mL of perchloric acid (0.05 M), mixed thoroughly, centrifuged (3,000 × *g* for 2 min), and supernatants recovered. Levels of calcium dissolved in supernatants were quantified using a flame photometer (Flame Photometer 410; Corning) calibrated using calcium standards at 100, 75, 50, and 25 ppm.

### Scanning electron microscopy of catheter cross sections

Biofilms formed on catheters recovered from timed models were visualized directly by scanning electron microscopy (SEM) as previously described by Holling *et al*.^20^. Catheter sections (1 cm) were mounted directly onto aluminum stubs using Leit adhesive carbon tabs (Agar Scientific, Stansted, United Kingdom), and stored overnight in a desiccator at room temperature. The following day, catheter sections were sputter coated with platinum using a Quorum Q150T ES system (Quorum Technologies, United Kingdom), and viewed using a Zeiss Evo LS15 microscope under high vacuum at an accelerating voltage of 5 kV and using a 5 quadrant backscatter detector (5Q-BSD).

### Statistical Analyses

All statistical analysis was performed using Prism 7.0c for Mac OS X (GraphPad Software, Inc., USA). Data were analyzed using either Student’s *t* test or analysis of variance with the Bonferroni correction of multiple comparisons.

## Electronic supplementary material

Supplementary Information
