# Supplementary material for: Fluoxetine and thioridazine inhibit efflux and attenuate crystalline biofilm formation by Proteus mirabilis
Source: Sci Rep. 2017 Sep 22;7:12222. doi: 10.1038/s41598-017-12445-w (PMC5610337; doi:10.1038/s41598-017-12445-w)
Supplement: Supplementary file 1 — Supplementary Information [file 41598_2017_12445_MOESM1_ESM.pdf]

Supplementary Information

Fluoxetine and thioridazine inhibit efflux and attenuate crystalline biofilm formation by *Proteus mirabilis*.

Nzakizwanayo JN[1#], Scavone P[1,2 #], Jamshidi S[3], Hawthorne JA[1], Pelling H[1], Dedi C[1], Salvage JP[1], Hind CK[4], Guppy FM[1], Barnes LM[1], Patel BA[1], Rahman KM[3], Sutton JM[4], Jones BV[1 *]

[1] School of Pharmacy and Biomolecular Sciences, University of Brighton, Lewes Road, Brighton, BN2 4GJ, United Kingdom

[2] Department of Microbiology, Instituto de Investigaciones Biológicas Clemente Estable, Montevideo CP 11600, Uruguay.

[3] Institute of Pharmaceutical Science, King's College London, 150 Stamford Street, London, SE1 9NH, United Kingdom

[4] National Infections Service, Public Health England, Porton Down, Salisbury, SP4 0JG, United Kingdom.

# These authors contributed equally

* Correspondence should be addressed to b.v.jones@brighton.ac.uk

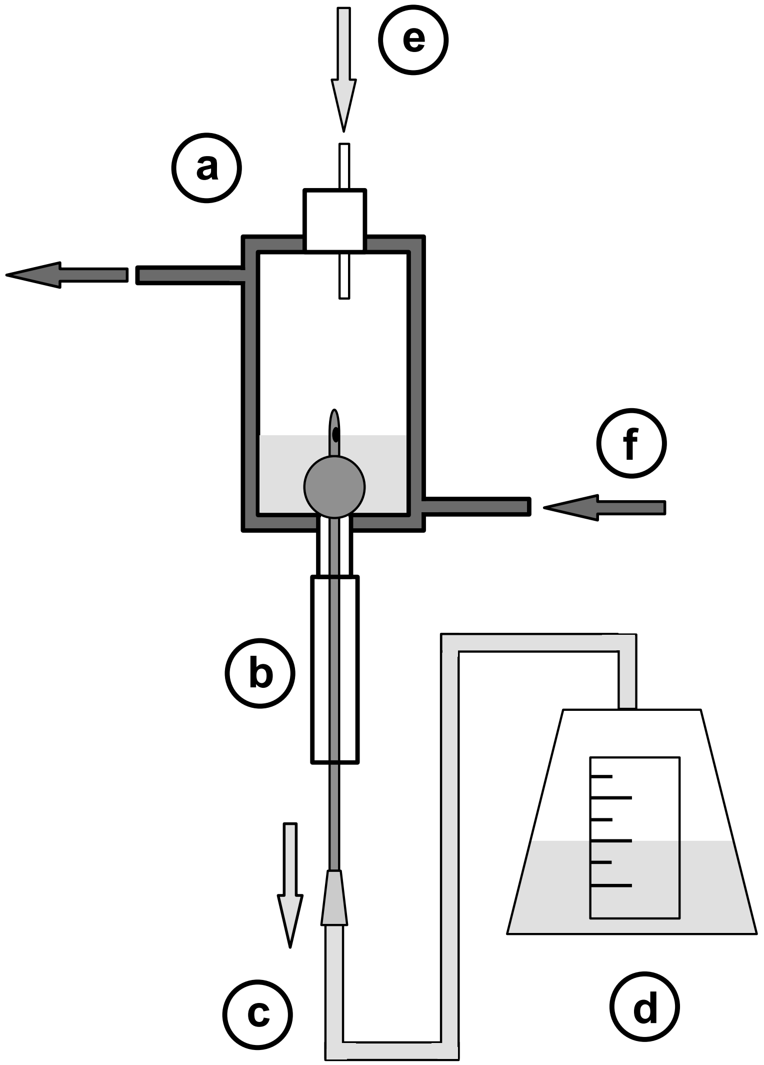

Supplementary Figure 1: Illustration of *in vitro* bladder model (from Nzakizwanayo *et al* 2016, *Antimicrobial Agents and Chemotherapy* 60:1530-1536**)**[27]**.** *In vitro* bladder models were set-up as described by Stickler *et al.* 1999. **a)** Double walled glass vessel representing the bladder. **b)** Foley catheter inserted into the model and connected to drainage bag to form sterile closed drainage system. **c)** Drainage tubing. **d)** Drainage bag collects urine outflow. **e)** Sterile urine/artificial urine supplied to "bladder" *via* peristaltic pump at a constant flow rate. **f)** Water at 37 ˚C circulated through outer bladder model chamber to maintain constant temperature.

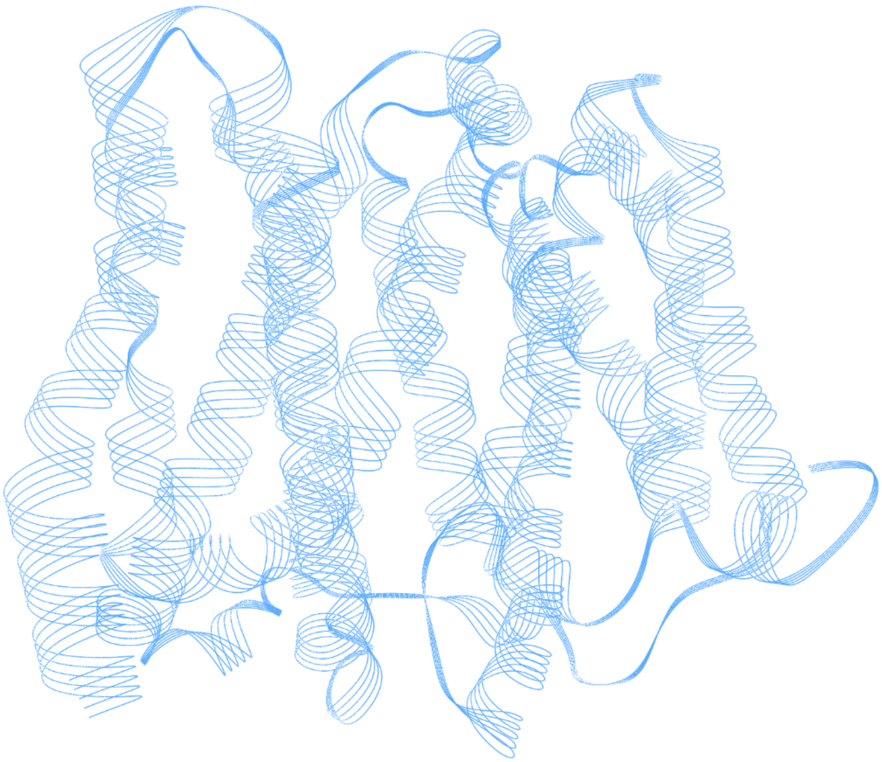

Supplementary Figure 2: 3D Homology model of *Bcr/CflA* efflux pump protein

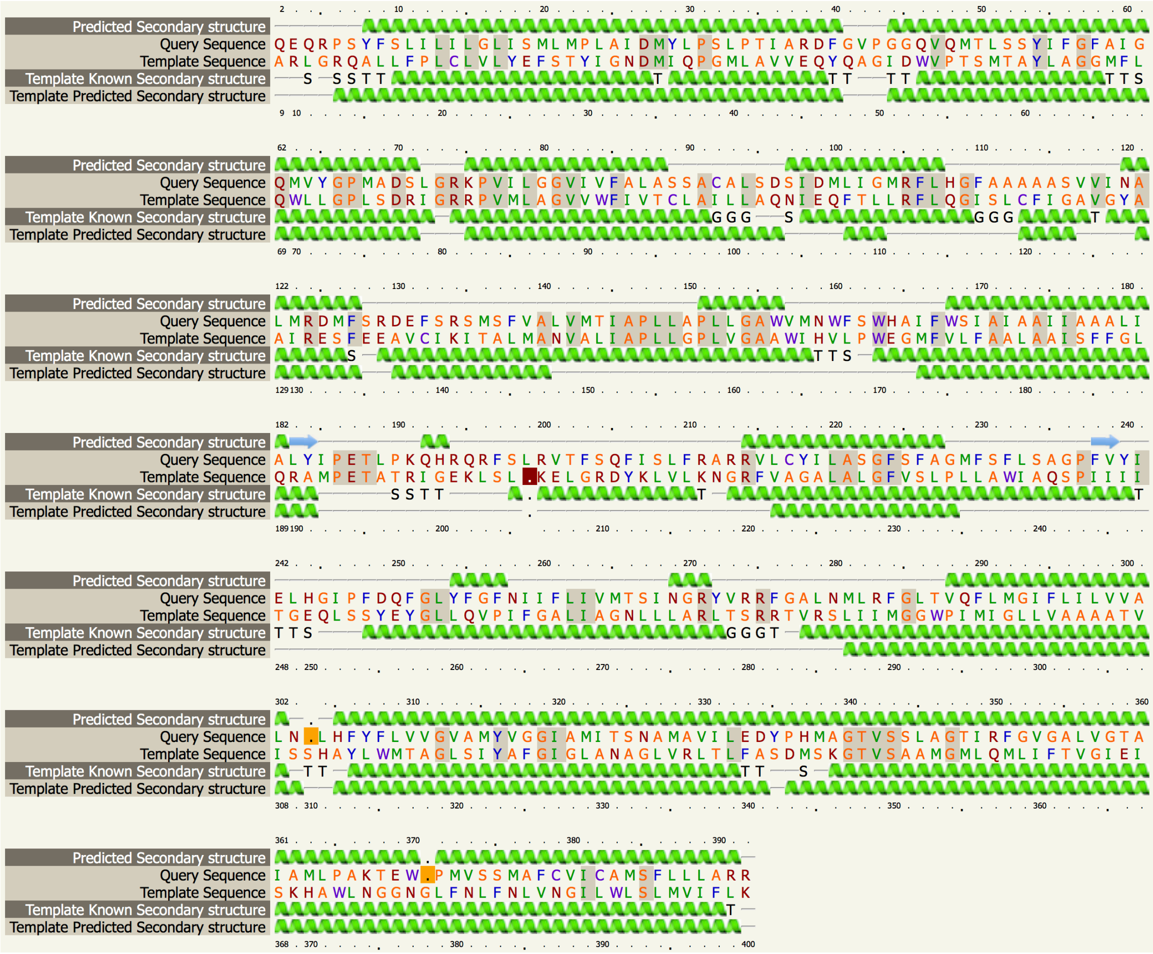

Supplementary Figure 3: Predicted secondary structure *Bcr/CflA* efflux pump. Original nucleotide sequence taken from the *P. mirabilis* HI4320 genome sequence (Pearson et al 2008, *J. Bacteriol.* 190:4027– 4037). Locus identifier: PMI_RS04070.

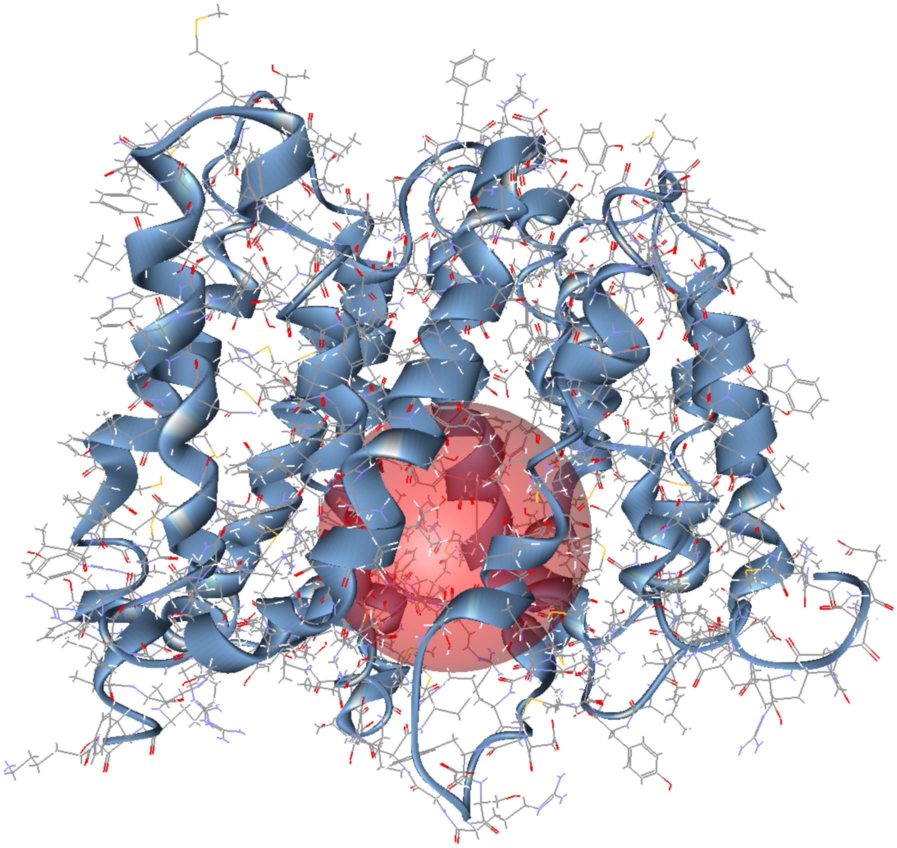

Supplementary Figure 4: Binding site of fluoxetine and thioridazine identified by SMINA molecular docking (Red sphere)

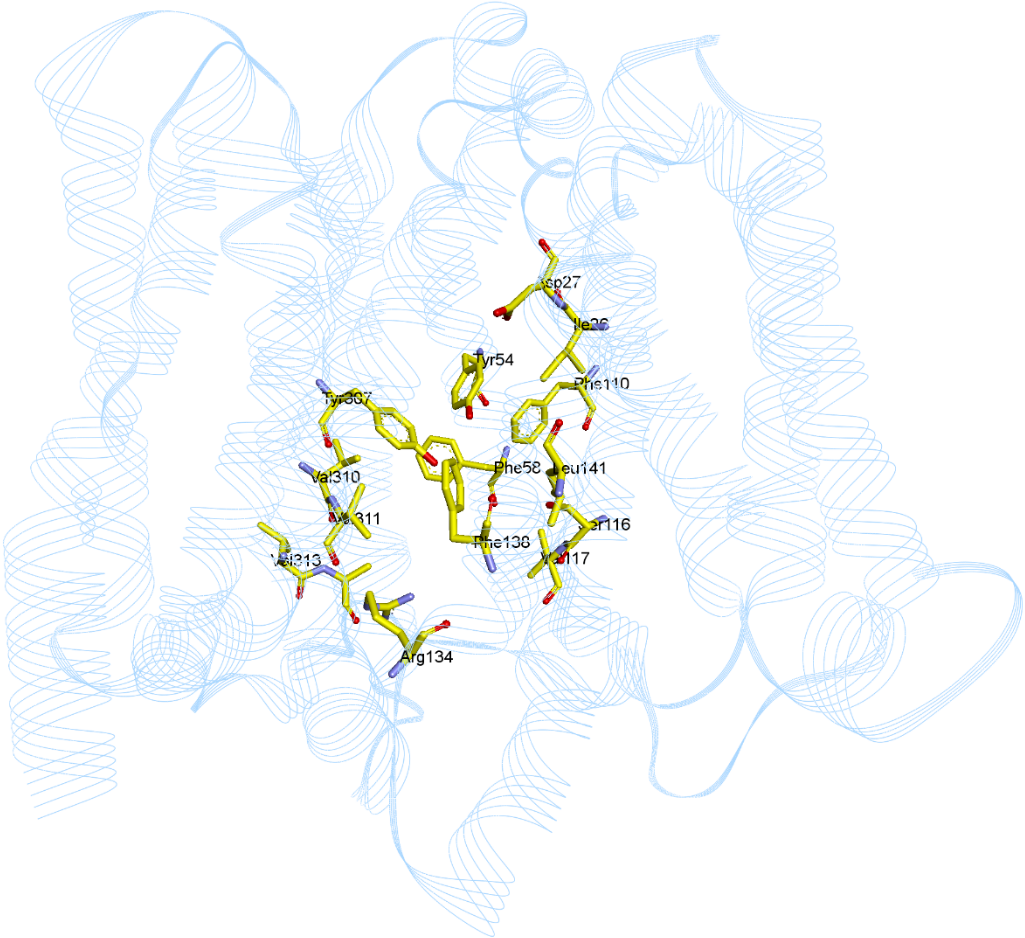

Supplementary Figure 5: Key residues of the binding site of *Bcr/CflA*

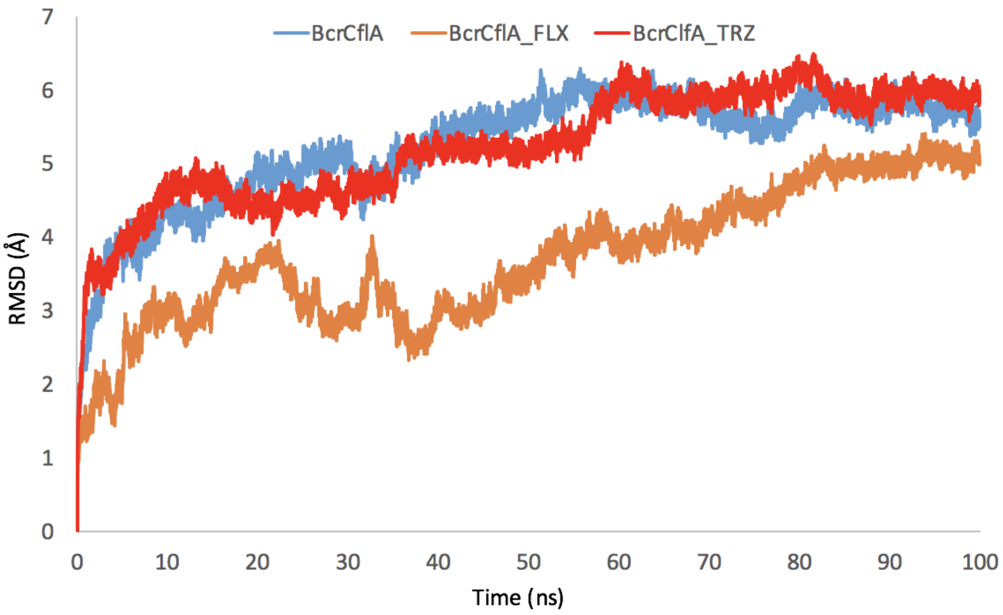

Supplementary Figure 6: The time dependence of root-mean-square deviation (RMSD) (Å) of *Bcr/CflA* pump over the Ca atoms (with respect to the internal structure) during the 100 ns MD simulations of both ligand-free and ligand-bound states. ***BcrCflA*** – no ligand; ***BcrCflA_FLX*** – with fluoxetine binding; ***BcrCflA_TRZ*** – with thioridazine binding
